# Matters arising: On the cost-effectiveness for the Italian National Health Service of nab-paclitaxel plus gemcitabine vs gemcitabine alone in metastatic pancreatic cancer

**DOI:** 10.1186/s42506-024-00179-3

**Published:** 2024-12-02

**Authors:** Carlo Lazzaro

**Affiliations:** 1 Studio di Economia Sanitaria, Via Stefanardo da Vimercate, 19, Milan, 20128 Italy; 2 Biology and Biotechnologies Department “Lazzaro Spallanzani”, University of Pavia, Pavia, 27100 Italy

## To the Editor,

In their interesting literature review on cost-effectiveness analysis thresholds [1], Fasseeh et al. [2] kindly included (as reference no. 88) an article I coauthored [3]. Following the Italian National Health Service (INHS) perspective [1], our article aimed at investigating the cost-effectiveness of nab-paclitaxel (Nab-P) + gemcitabine (G) vs G alone in metastatic pancreatic cancer (MPC) via a 4-year, four health states (progression free, progressed, end of life, death), Markov model supported cost-utility analysis [1, 4].

Being extremely grateful for Fasseeh and colleagues’ interest in our research, I would like to clarify some points that their article raises.

Table 2 (page 6) [2] of Fasseeh and colleagues’ article reports an unofficial 25,000 €–45,000 € acceptability range for incremental life year saved [1] or quality-adjusted life years (QALYs) gained [1] for Italy.

While the lower limit of the range is correct, the upper one is not.

In fact, the Italian guidelines for the economic evaluation of health care programmes proposed by the Italian Association for Health Economics (Associazione Italiana di Economia Sanitaria — AIES) suggest an upper limit of 40,000 € (2008 values) for that range [5, 6]. Remarkably, the informal 25,000 €–40,000 € range suggested by AIES [5, 6] represents an implicit conversion in € of the official UK £25,000–UK £40,000 range adopted by the National Institute for Health and Clinical Excellence [7].

As mentioned in our article (pages 440–441) [3], an informal 87,330 € threshold value (95% confidence interval: 37,024 € and 137,636 €) was retrospectively calculated for Italy on the grounds of the reimbursement decisions made by the Italian Medicines Agency concerning 32 cancer therapies that entered the Italian market during 2010–2013 [8]. We compared its sample estimate (87,330 €) to our baseline incremental cost-utility ratio of Nab-P + G (46,021.58 €, 2017 values; incremental cost: 7082.68 €; incremental QALYs: 0.154) and proved the cost-effectiveness for the INHS of Nab-P + G vs G alone in patients with MPC.

Conversely, it remains unclear where Fasseeh and colleagues (page 9) [2] found in our article the 30,000 € threshold that they attribute to our research.

In this era of increasing number of scientific journals that can publish a theoretically boundless volume of articles online, drafting a sound and informative literature review is getting more and more difficult and time-consuming. Even though the selected contributions are consistent with the research question, double-checking all the details of each paper is often unfeasible.

Whenever the research topic includes national guidelines, a further effort aimed at skimming through the original sources is warranted. If the guidelines are not available in English, researchers are recommended to ask the corresponding author for clarifications.

Other things being equal, this approach is expected to increase the quality of literature reviews.

## Data Availability

No datasets were generated or analysed during the current letter.

## Abbreviations

AIES: Associazione Italiana di Economia Sanitaria (Italian Association for Health Economics)
G: Gemcitabine
INHS: Italian National Health Service
MPC: Metastatic pancreatic cancer
Nab-P: Nab-paclitaxel
QALY: Quality-adjusted life year
